# Advancing stroke therapy: innovative approaches with stem cell-derived extracellular vesicles

**DOI:** 10.1186/s12964-024-01752-1

**Published:** 2024-07-22

**Authors:** Jiahao Song, Da Zhou, Lili Cui, Chuanjie Wu, Lina Jia, Mengqi Wang, Jingrun Li, Jingyuan Ya, Xunming Ji, Ran Meng

**Affiliations:** 1 Department of Neurology, Xuanwu Hospital, Capital Medical University, Beijing, 100053 China; 2 Advanced Center of Stroke, Beijing Institute for Brain Disorders, Beijing, 100053 China; 3 National Center for Neurological Disorders, Xuanwu Hospital, Capital Medical University, Beijing, 100053 China; 4 Academic Unit of Mental Health and Clinical Neuroscience, School of Medicine, University of Nottingham, Nottingham, England

**Keywords:** Stroke, Stem cell-derived, Extracellular vesicles, Therapeutic approach, Neuroprotection

## Abstract

Stroke is a leading cause of mortality and long-term disability globally, with acute ischemic stroke (AIS) being the most common subtype. Despite significant advances in reperfusion therapies, their limited time window and associated risks underscore the necessity for novel treatment strategies. Stem cell-derived extracellular vesicles (EVs) have emerged as a promising therapeutic approach due to their ability to modulate the post-stroke microenvironment and facilitate neuroprotection and neurorestoration. This review synthesizes current research on the therapeutic potential of stem cell-derived EVs in AIS, focusing on their origin, biogenesis, mechanisms of action, and strategies for enhancing their targeting capacity and therapeutic efficacy. Additionally, we explore innovative combination therapies and discuss both the challenges and prospects of EV-based treatments. Our findings reveal that stem cell-derived EVs exhibit diverse therapeutic effects in AIS, such as promoting neuronal survival, diminishing neuroinflammation, protecting the blood-brain barrier, and enhancing angiogenesis and neurogenesis. Various strategies, including targeting modifications and cargo modifications, have been developed to improve the efficacy of EVs. Combining EVs with other treatments, such as reperfusion therapy, stem cell transplantation, nanomedicine, and gut microbiome modulation, holds great promise for improving stroke outcomes. However, challenges such as the heterogeneity of EVs and the need for standardized protocols for EV production and quality control remain to be addressed. Stem cell-derived EVs represent a novel therapeutic avenue for AIS, offering the potential to address the limitations of current treatments. Further research is needed to optimize EV-based therapies and translate their benefits to clinical practice, with an emphasis on ensuring safety, overcoming regulatory hurdles, and enhancing the specificity and efficacy of EV delivery to target tissues.

**Graphical Abstract:**

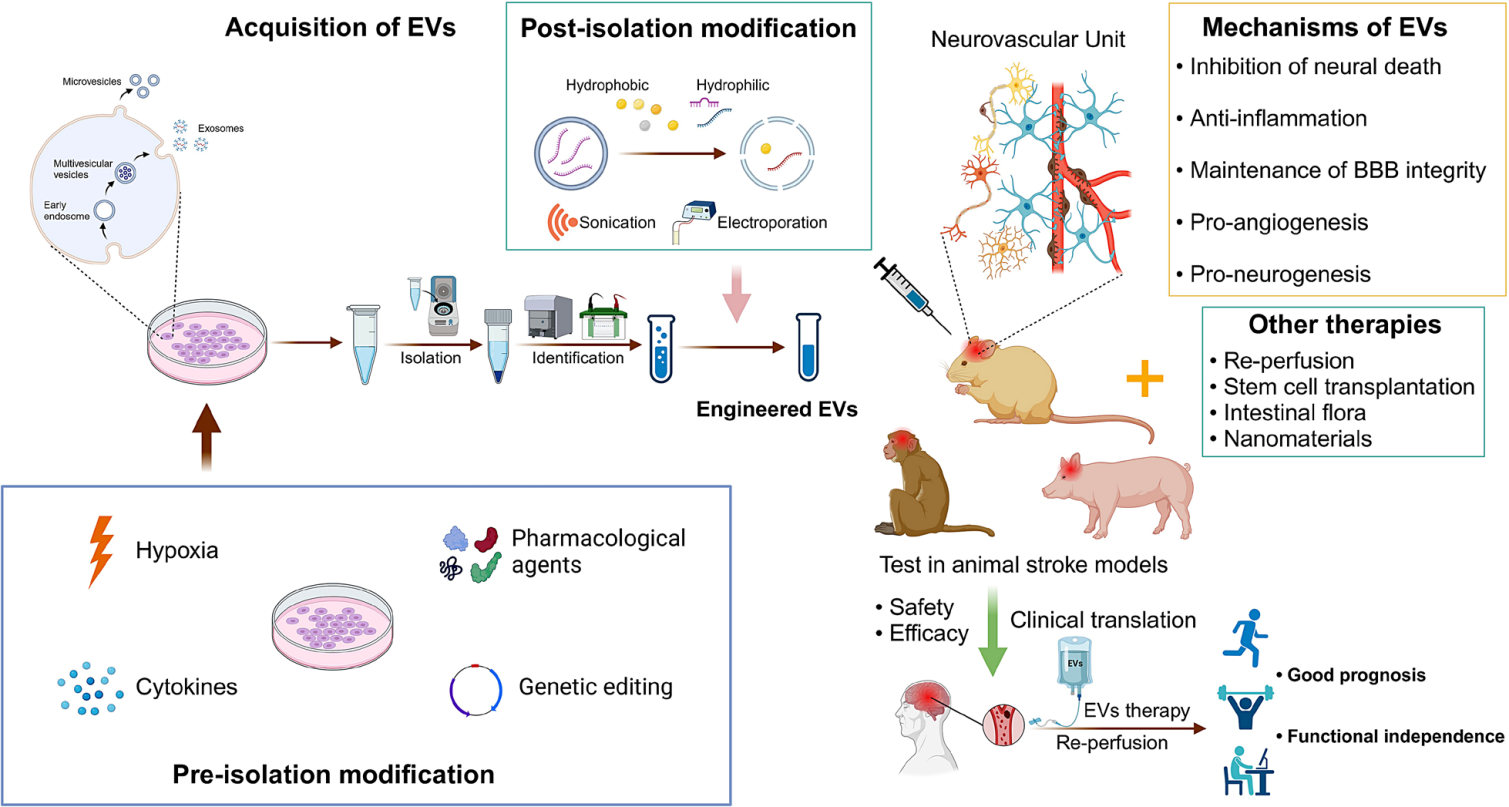

## Introduction

**Fig. 1:**
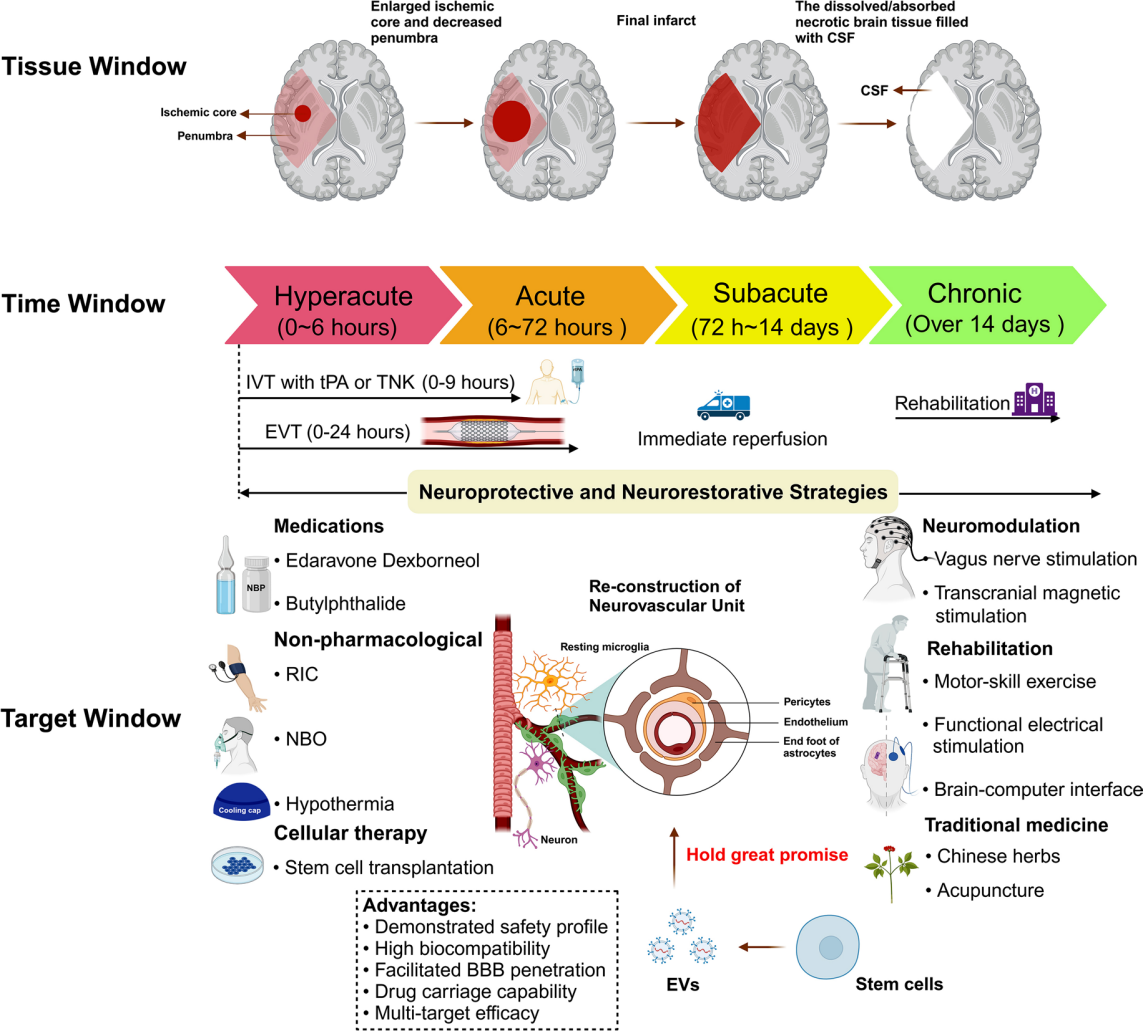
Evolving concepts of reperfusion therapy for AIS and current adjunctive strategies for neuroprotection. The advent of multimodal neuroimaging techniques has revolutionized stroke management by enabling the estimation of perfusion mismatch and providing insights into the volume of brain tissue amenable to salvage, shifting the focus from a rigid time-based approach to a more dynamic tissue window-guided strategy. In addition to reperfusion therapy with IVT and EVT, additional neuroprotective strategies are anticipated to target multiple aspects of the neurovascular unit after ischemic cascades over time. EVs derived from stem cells are increasingly recognized as a viable treatment option for stroke due to their inherent attributes, including demonstrated safety profile, high biocompatibility, the ability to traverse biological barriers, drug carriage capability, and multi-target efficacy. Abbreviations: AIS = acute ischemic stroke; IVT = intravenous thrombolysis; EVT = endovascular thrombectomy; EVs = extracellular vesicles; CSF = cerebrospinal fluid; tPA = alteplase; TNK = tenecteplase; RIC = remote ischemic conditioning; NBO = normobaric oxygen; BBB = blood-brain barrier. Figure created with BioRender.com

According to the Global Burden of Disease analysis, stroke ranks as the second leading cause of death and the third leading cause of combined death and disability worldwide [1]. Acute ischemic stroke (AIS), the most prevalent subtype, poses a substantial threat to global health and economics [1, 2]. Current standard treatments for AIS, including intravenous thrombolysis (IVT) with alteplase (tPA) and endovascular thrombectomy (EVT), are hampered by their limited therapeutic windows—4.5 to 9 h for IVT and 6 to 24 h for EVT [3]—and associated risks such as hemorrhagic transformation [4]. These constraints render them unsuitable or ineffective for a significant patient cohort. Indeed, data from a national register in New Zealand revealed that only 13.4% of AIS patients received IVT or EVT, with merely half achieving functional independence after three months [5, 6], underscoring the urgent need for novel therapeutic strategies. Figure 1 depicts evolving concepts of reperfusion therapy for AIS and current adjunctive strategies for neuroprotection.

Stem cell-based therapies, utilizing mesenchymal stem or stromal cells (MSCs), neural stem cells (NSCs), and induced pluripotent stem cells (iPSCs), have emerged as promising avenues due to their potential in neuroprotection and neurorestoration [7, 8]. These therapies principally operate through two mechanisms. Initially, stem cells act as ideal “seed cells” that differentiate into neuronal and glial cells to replace those lost to ischemia. Studies have shown that within 30 days post-transplantation of human MSCs, there is a significant upregulation of neural-specific and synapse-associated proteins, as well as human-specific markers [9], signifying the successful differentiation of the transplanted MSCs. Additionally, the paracrine effects, primarily mediated through the secretion of extracellular vesicles (EVs), cytokines, chemokines, and neurotrophic factors, are vital for therapeutic efficacy following transplantation [10]. However, the practical application of these cell-based approaches is challenged by the low survival rates of transplanted cells in the hostile post-ischemic environment, where less than 1% survive and differentiate in vivo [11, 12], and additional concerns such as the risk of embolism, unpredictable differentiation pathways, and storage difficulties [13].

Consequently, research focus has shifted towards the paracrine capabilities of stem cells, particularly the role of EVs. Stem cell-derived EVs, abundant in bioactive molecules, facilitate intercellular communication and possess immunomodulatory and tissue restorative properties akin to their cells of origin. Their inherent attributes, including immunological inertness, low cytotoxicity, and the capacity to traverse biological barriers [14], along with the possibility of targeted and cargo modifications, position them as a viable cell-free therapeutic option [15]. Thus, EV-based therapies have garnered considerable interest in pre-clinical studies for AIS, heralding a new era in the quest for effective stroke treatments. This review meticulously summarizes up-to-date research concerning the use of EVs in AIS therapy, emphasizing their origin, biogenesis, mechanisms of action, and strategies to enhance their targeting accuracy and therapeutic potential. Furthermore, it outlines innovative combinations with other novel treatments, acknowledges existing challenges, and proposes future directions for the clinical application of EVs in stroke therapy.

## Pathophysiology of AIS

**Fig. 2:**
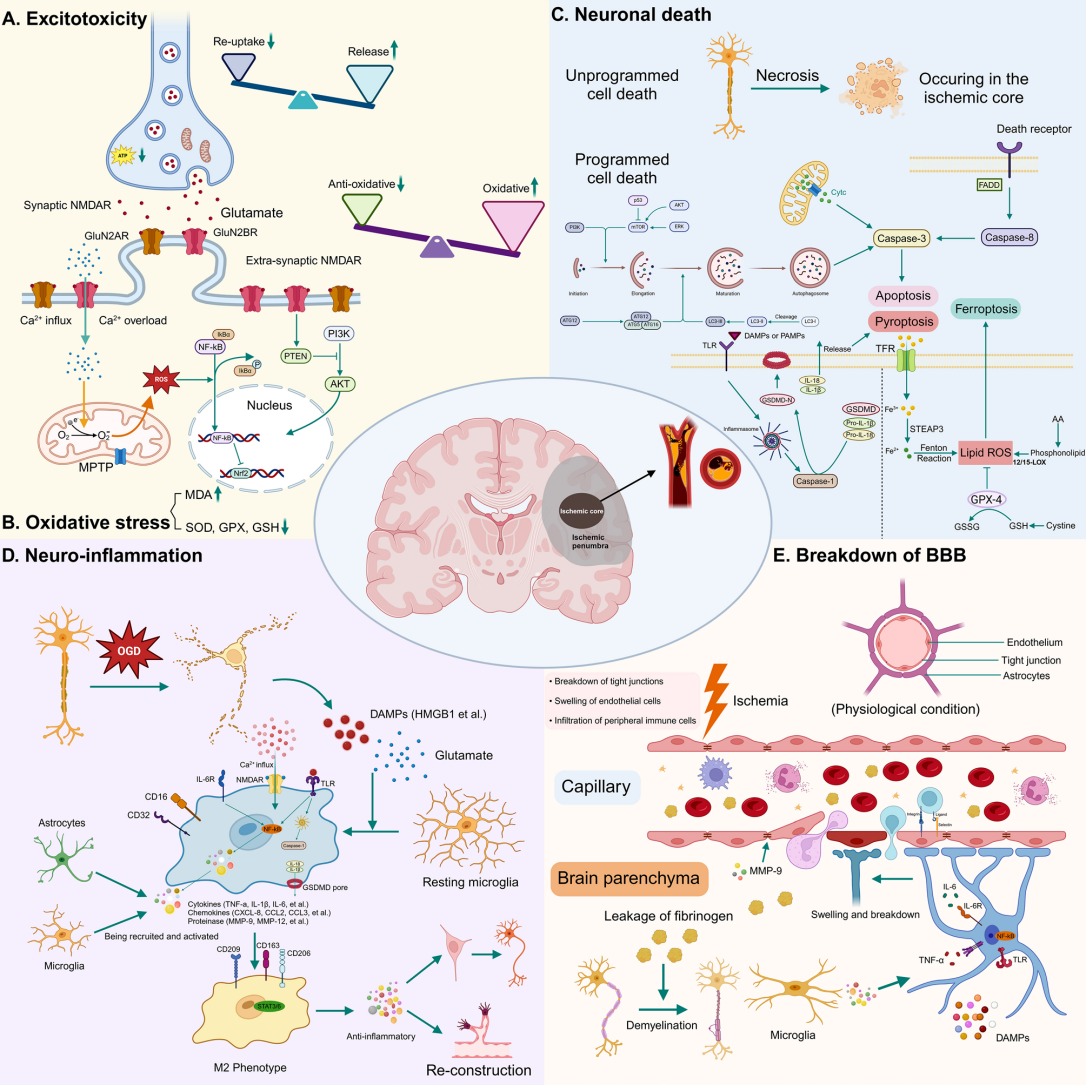
Pathophysiological processes in AIS. **(A)** Excitotoxicity: Under OGD, the balance between the release and re-uptake of glutamate is disrupted, resulting in the accumulation of glutamate and subsequent calcium ion influx into neurons. **(B)** Oxidative stress: Stimulated by ischemia and calcium overload, mitochondrial dysfunction occurs and large quantities of ROS are released. **(C)** Neuronal death: Neurons initiate autophagy to adapt to harsh conditions, but over-activation of autophagy may trigger apoptosis through various pathways. Additionally, pyroptosis and ferroptosis are critical cell death mechanisms for neurons under OGD. **(D)** Neuroinflammation: In the acute phase, microglia, peripheral macrophages, and astrocytes are activated and release a variety of pro-inflammatory cytokines. In the chronic phase, neuroinflammation is significant for later neuro-restoration. **(E)** Loss of BBB integrity: Infiltration of peripheral immune cells, direct injury from OGD, and swelling and death of astrocytes all contribute to BBB disruption. Abbreviations: AIS = acute ischemic stroke; OGD = oxygen glucose deprivation; ROS = reactive oxygen species; BBB = blood brain barrier; NMDAR = N-methyl-D-aspartate receptor; MDA = malondialdehyde; SOD = superoxide dismutase; GPX = glutathione peroxidase; GSH = glutathione; DAMPs = damage-associated molecular patterns; PAMPs = pathogen-associated molecular patterns; TLR = Toll-like receptor; TFR = transferrin receptor; AA = arachidonic acid; GSSG = glutathione disulfide; HMGB-1 = high mobility group protein; FADD = Fas-associating protein with a novel death domain; LOX = lipoxygenase; GSDMD = gasdermin D; IL = interleukin; CXCL = C-X-C motif chemokine ligand; CCL = C-C motif chemokine ligand; TNF-α = tumor necrotic factor-α; IL-6R = interleukin-6 receptor; MMP = matrix metalloproteinase. Figure created with BioRender.com

Cerebral vessel occlusion leads to oxygen and glucose deprivation (OGD) in focal brain tissues, precipitating metabolic distress in neurons and glial cells [16, 17]. The primary pathophysiological processes in AIS include oxidative stress, excitotoxicity, neural death, neuroinflammation, and loss of blood-brain barrier (BBB) integrity. Sudden OGD causes mitochondrial dysfunction, characterized by ATP scarcity, which results in electron leakage from the electron transport chain and excessive production of reactive oxygen species (ROS) [18, 19]. Concurrently, increased mitochondrial membrane permeability facilitates the release of apoptosis-inducing proteins like cytochrome C from mitochondrial permeability transition pores, triggering intrinsic-pathway cell apoptosis [19, 20]. Under ATP depletion, the function of the sodium pump on neuronal surfaces is inhibited, resulting in cell membrane depolarization and subsequent release of large amounts of excitatory neurotransmitters. Furthermore, neurons and surrounding glial cells are unable to re-uptake these substances promptly. This results in a notable accumulation of glutamate, which over-activates NMDA receptors, especially extra-synaptic ones, and induces calcium overload [21] (Fig. 2A). On one hand, excessive calcium influx triggers proteases that destroy cellular structures. On the other hand, calcium overload exacerbates mitochondrial dysfunction, further enhancing ROS production (Fig. 2B). Additionally, activation of extra-synaptic NMDA receptors can activate downstream PTEN, which inhibits the PI3K/Akt signaling pathway and triggers cellular death [22].

Direct injury from OGD, accumulation of ROS, excitotoxicity, and mitochondrial dysfunction ultimately inhibit cell viability and induce neuronal death. Compared to the necrotic process in the ischemic core, programmed cellular death—including apoptosis, pyroptosis, ferroptosis, and autophagy—warrants more attention. Under OGD, neurons initiate autophagy to clear certain organelles and proteins, reducing metabolic levels to adapt to harsh conditions. Nevertheless, excessive autophagy can activate Caspase-3, triggering apoptosis. Additionally, pyroptosis, characterized by the assembly of the NLRP-3 inflammasome, and ferroptosis, marked by the accumulation of lipid ROS, also play significant roles in neuronal death (Fig. 2C).

Neuroinflammation is a critical component of AIS pathophysiology and spans the entire disease process. Initially, damage-associated molecular patterns from injured cells provoke peripheral immune cell migration to the infarct zone and activate microglial transformation to the M1 phenotype [23]. Newly recruited inflammatory cells, including macrophages, T cells, M1 microglia, and astrocytes, release a variety of cytokines, chemokines, and matrix metalloproteinases (MMPs), which further magnify the destructive process. Following the acute phase, a reduction in peripheral immune infiltration and a shift toward anti-inflammatory cytokines promote angiogenesis and neural remodeling, marking the commencement of the repair and recovery phase [24] (Fig. 2D).

In addition to neurons, ischemic events also compromise endothelial cells, tight junctions, and astrocytes, undermining BBB integrity [25], a predictor of adverse outcomes and hemorrhagic transformation in AIS [26, 27]. Pro-inflammatory agents, astrocyte injury, peripheral macrophage infiltration, and the proteolytic effects of MMP further disrupt BBB integrity and exacerbate leakage. Fibrinogen from plasma can damage oligodendrocytes and result in demyelination (Fig. 2E). Given the dynamic and complicated nature of post-stroke pathophysiology, integrating neuroprotective and neurorestorative interventions with reperfusion therapies is imperative. Effective therapeutic strategies should aim to target the salvageable ischemic penumbra to mitigate neuronal loss and inflammation while supporting angiogenesis and neural remodeling throughout the acute to chronic recovery phases [28–31].

## Extracellular vesicles (EVs)

### Overview of EVs

EVs, encapsulated by bilayered phospholipid membranes, are ubiquitously present in various bodily fluids, including plasma, urine, saliva, semen, breast milk, and cerebrospinal fluid. These vesicles, secreted by an extensive array of cellular types, were initially identified in 1983 by C. Harding as alternative vehicles for cellular waste disposal [32, 33]. Subsequent research, notably by G. Raposo et al. in 1996, uncovered their role in antigen presentation, thus expanding our understanding of their diverse biological function [34]. Recent advances have further emphasized the critical role of EVs in mediating intercellular communication and modulating cell-extracellular matrix interactions, underpinning their significance in both physiological and pathological contexts [35, 36]. Notably, EVs display considerable heterogeneity in structure and composition and are primarily categorized into exosomes, microvesicles (MVs), and apoptotic bodies (ApoBDs) based on their size, density, and biogenesis process [37, 38]. Exosomes typically measure 30–150 nm and have a density of 1.13–1.19 g/ml. MVs generally range from 50 to 1,000 nm with densities of 1.16–1.19 g/ml, while ApoBDs are larger, ranging from 50 to 5,000 nm, and denser, with densities of 1.16–1.28 g/ml [39–41]. Among these, exosomes have been the focus of extensive research. However, due to the absence of standardized methods to precisely distinguish between EV types, those isolated from cell culture media are often a heterogeneous mixture, predominantly composed of exosomes.

### Origin and Biogenesis of EVs

**Fig. 3:**
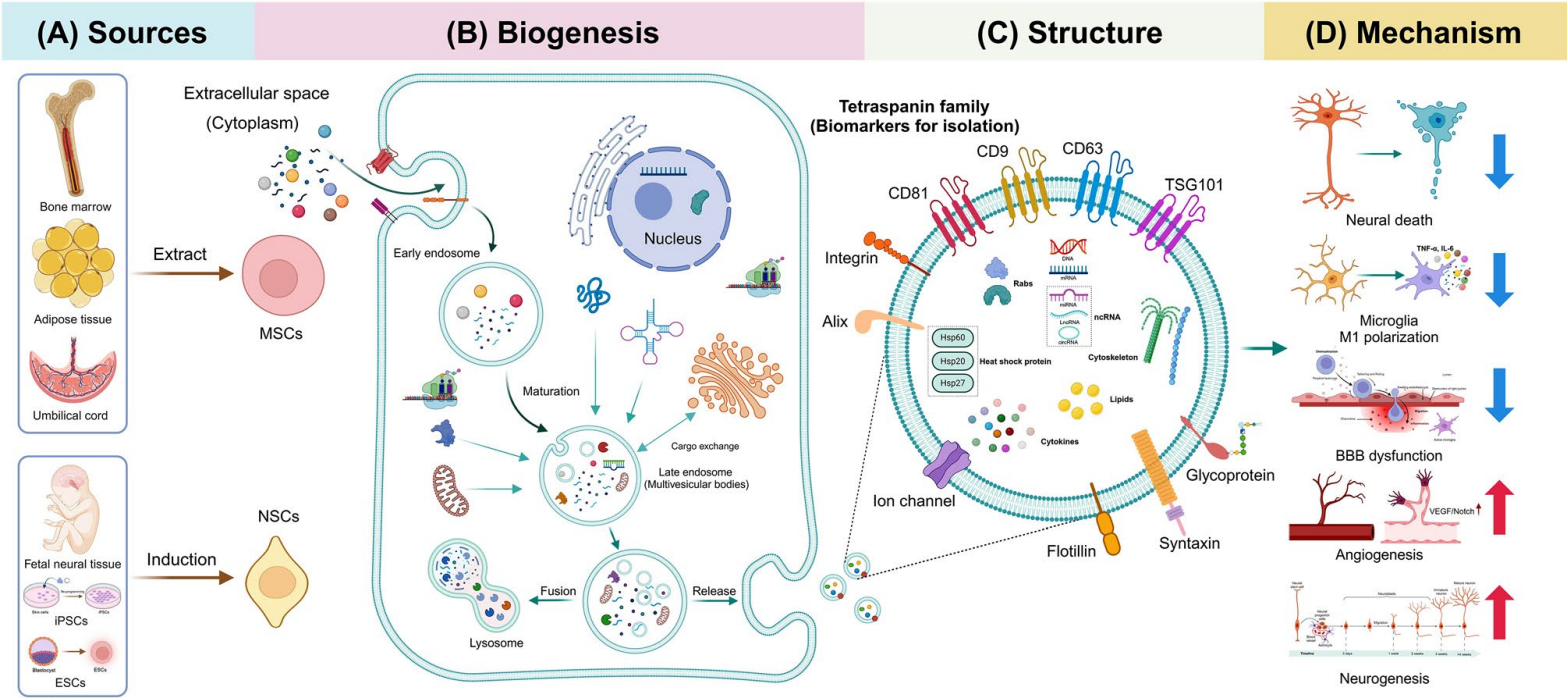
Biological sources, biogenesis, and structure of stem cell-derived exosomes as well as their potential mechanisms in treating AIS. **(A)** Exosomes utilized in AIS treatment are primarily sourced from MSCs and NSCs. **(B)** The biogenesis process of exosomes involves a two-stage endocytosis process. The initial invagination of the plasma membrane encapsulates extracellular substances to form EEs. Subsequent inward budding of the EEs’ membrane, in tandem with material exchange with other cell organelles, leads to the formation of LEs or MVBs. Upon fusing with the plasma membrane, exosomes are discharged into the extracellular milieu from MVBs to manifest their biological functions. **(C)** Exosomes possess a complex architecture, comprising a diverse array of lipids, proteins, and nucleic acids. **(D)** Exosomes exhibit multifaceted therapeutic properties in treating AIS, including the inhibition of neuronal apoptosis, reduction of microglial cell activation, protection against BBB breakdown, and facilitation of angiogenesis and neurogenesis. Abbreviations: AIS = acute ischemic stroke; MSCs = mesenchymal stem cells; NSCs = neuronal stem cells; EEs = early endosomes; LEs = late endosomes; MVBs = multivesicular bodies; BBB = blood-brain barrier; iPSCs = induced pluripotent stem cells. Figure created with BioRender.com

EVs originate from an array of cell types, with a focus here on those derived from stem cells, specifically MSCs and NSCs, due to their relevance in AIS treatment [42, 43]. MSCs, known for their self-renewal capabilities and pluripotency, can differentiate into both mesenchymal and non-mesenchymal tissue lineages under appropriate conditions [44]. These cells are harvested from diverse biological sources, including adipose tissue, bone marrow, fetal placenta, umbilical cord, and urine [45–47]. NSCs, albeit more challenging to extract, are typically located in brain niches like the subventricular zone (SVZ) and subgranular zone (SGZ) [48]. Alternative sources such as embryonic stem cells (ESCs) and iPSCs also provide NSCs, with iPSCs introduced by Takahashi and Yamanaka in 2007 circumventing ethical concerns associated with ESCs [49–51] (Fig. 3A).

The biogenesis of EVs, particularly exosomes, entails a series of endocytosis and membrane fusion events [52]. Originating from the endosomal system, exosomes develop through the inward budding of the plasma membrane to form early endosomes, which mature into multivesicular bodies (MVBs). A key step in this process involves the sorting of cargo into intraluminal vesicles (ILVs) within MVBs, orchestrated by the Endosomal Sorting Complex Required for Transport (ESCRT) machinery [53]. In 2008, a notable study by K. Trajkovic et al. identified an ESCRT-independent pathway involving ceramides for MVB maturation [54]. ILVs can either be released as exosomes or degraded upon fusion of MVBs with lysosomes or autophagosomes [55] (Fig. 3B). The trafficking and plasma membrane fusion of these vesicles involve actin and microtubule scaffolds and Rab small GTPases [56, 57]. In contrast, MVs are generated by the direct outward budding of the plasma membrane, while ApoBDs result from apoptotic cells through processes involving cellular contraction and membrane blebbing [58], each playing distinct roles in immunoregulation, disease diagnostics, and regenerative therapy [59, 60].

### Components of EVs

EVs are versatile carriers, harboring a variety of biological materials that reflect their formation processes. Exosomes are particularly noted in therapeutic contexts for their rich cargo, as cataloged in the ExoCarta database (http://www.ExoCarta.org/), which lists 41,860 proteins, 4,946 messenger RNAs (mRNAs), 2,838 microRNAs (miRNAs), and 1,116 lipids. Some stem cell-derived exosomes can even transport functional mitochondria, highlighting their potential in regeneration [61, 62]. Heat shock proteins, cytoskeletal proteins, growth factors, cytokines, ESCRT machinery-associated proteins (e.g., Alix, TSG101), and membrane transport and fusion proteins collectively constitute the intricate protein network within exosomes. The composition of exosomes varies with the donor cell type, cultivation conditions, and isolation methods, yet certain proteins like Alix and TSG-101, are consistently present and integral to MVB formation [63].

Non-coding RNAs (ncRNAs) within exosomes, such as miRNAs, long non-coding RNA (lncRNAs), and circular RNAs, play crucial roles in modulating gene expression and influencing recipient cell functions. A plethora of studies have elucidated that ncRNAs are potential targets for stroke therapy, possibly by the regulation of processes involving neuroinflammation, cellular injury, BBB dysfunction, and oxidative stress [64, 65]. Moreover, the integration of gene-editing techniques to incorporate exogenous ncRNAs into exosomes represents a promising strategy for enhancing drug encapsulation and delivery.

Apart from cargo, the phospholipid bilayer membrane of exosomes houses essential proteins and lipids that ensure membrane stability and functionality. Among these, the tetraspanin family proteins, especially CD63, are notable for their roles in exosome formation, cargo recruitment, and assembly [66]. These elements are integral to the identification and therapeutic application of exosomes [67] (Fig. 3C).

### Isolation and purification

The isolation and purification of EVs, particularly exosomes, from stem cell culture media are paramount for their thorough study and therapeutic application. Techniques for EV separation primarily hinge on the physicochemical properties of EVs, including density, size, molecular weight, surface charge, solubility, and immunological biomarkers [68].

Ultracentrifugation serves as a conventional approach to isolate EVs utilizing centrifugal force. Differential and density gradient ultracentrifugation are commonly used subtypes. Differential ultracentrifugation, the gold-standard method, involves multiple centrifugation steps to eliminate cells and debris before isolating EVs at very high speeds [69]. Initially, cells and cellular components are removed during centrifugation at 300–400 g. Subsequently, cellular debris and bio-polymers are separated at 2,000 and 10,000 g, respectively. Ultimately, EVs (mainly exosomes) are sedimented under high speeds (usually 100,000-200,000 g) for approximately two hours. Despite its widespread use, this method is often criticized for its inefficiency, long duration, and labor-intensive nature.

Density gradient centrifugation offers an effective alternative by creating a centrifuge tube density gradient to separate EVs. A continuous density gradient is formed, allowing EVs to remain suspended at their appropriate density layer during ultracentrifugation. Sucrose and iodixanol are the primary media used to create this gradient. However, this method requires precise setup and professional operators, and is less practical for routine use [70].

Size-based purification techniques like ultrafiltration and size-exclusion chromatography (SEC) are also utilized. Ultrafiltration, facilitated by nanoporous membranes and pressure differential, separates EVs based on size. Typically, centrifugation and filtration are used in tandem, where the stem cell culture medium is first centrifuged to remove cellular contaminants and prevent the blockage of filtering membranes, followed by filtration and another centrifugation step to collect EVs [71]. Although effective, filtration may lead to membrane clogging and potential damage to EV structure. SEC achieves EV separation using porous spherical beads. When the bio-sample containing EVs is added to the SEC column, smaller particles are captured in the pores, while larger molecules, unable to enter the pores, are eluted first. Consequently, smaller molecules are retained in the pores and require more time to be eluted. SEC provides more precise size separation and minimizes membrane damage, but it is challenging to scale for clinical use due to purity concerns [72].

Polymer precipitation separates EVs by adding hydrophilic polymers to the bio-sample, reducing the hydrophilicity and solubility of EVs. Polyethylene Glycol (PEG) is the most widely used polymer, enabling simpler and faster isolation of EVs. However, it risks co-isolating non-vesicular contaminants, which can adversely affect the purity and functionality of the isolated EVs [37]. Affinity-based separation exploits specific surface proteins of EVs, using immunomagnetic beads that target tetraspanins (e.g., CD63, CD9, CD81). This method allows for high-purity isolation but poses challenges in removing bound antibodies without compromising EV integrity [73].

The advent of microfluidic technologies presents a promising avenue for enhancing substance separation. These technologies manipulate minuscule fluid volumes through microchannels with diameters ranging from tens to hundreds of microns. When integrated with traditional separation techniques, microfluidics can boost EV yield and purity. Innovations in microfluidic chips, which incorporate elements such as antibodies, nanomembranes, and magnetic nanobeads, have shown potential, though their application is limited by the need for specialized equipment [74]. More details about the comparisons of different EV isolation methods are listed in Table 1.

**Table 1:** **Summary of techniques utilized for the isolation and purification of EVs**

Technique	Principle	Material	Advantages	Disadvantages	Reference
Differential ultracentrifugation	Size and density	Centrifugal machine	No requirement for special reagents; Simplicity of operation; Suitable for large-scale samples	Time-consuming; Labor-intensive; Relatively low purity; Unstable recovery rate; Potential damage to EVs from repeated centrifugation; High equipment requirements	[69]
Density gradient centrifugation	Density	Medium of density gradient (sucrose and iodixanol)	Relatively high purity; Suitable for large-scale samples	Time-consuming and complicated steps; Damage to EVs from repeated centrifugation; High equipment requirements	[70]
Ultrafiltration	Size	Filtration membranes	Simplicity and efficiency; No need for high-precision equipment	Clogging of the filtration membrane by EVs; Reduced membrane lifespan; Adhesion of EVs on filtration membranes and difficulty to harvest	[71]
Size exclusion chromatography	Size	Porous polymer gel	High efficiency; High purity	Suitable only for small-volume bio-samples; Requirement for special equipment	[72]
Polymer precipitation	Hydrophilicity and solubility	Hydrophilic polymers (PEG, lectin, et al.)	Simple operation; Short-time consumption; No special equipment requirement	Low purity; Susceptibility to impurity proteins and polymers; Damage of EVs	[37]
Affinity-based isolation	Antigen-Ab interaction	Immunomagnetic beads	High purity; High specificity; Simplicity of operation; No need for special equipment	High cost; Unsuitability for large-scale production; Susceptibility of EVs’ activity and structure to later separation procedures	[73]
Microfluidic-based isolation	Mechanical agitation, immunological affinity, and electromagnetic field	Microfluidic system	High purity; High efficiency; Automation	Costly; Requirement of special equipment; Lack of validation and standardization	[74]

Abbreviations EVs = extracellular vesicles; PEG = polyethylene glycol; Ab = antibody

### Identification and characterization

Following the isolation of EVs, accurate identification and characterization are vital for determining their dosage, quality control, and clinical utility [75]. Employing a suite of complementary techniques ensures a comprehensive evaluation of EV purity. Key aspects of EV characterization include their morphological structure, size and distribution, cellular origin, and biological composition. Transmission electron microscopy is extensively used to analyze EV morphology and size, typically revealing the characteristic round, oval, or cup-shaped forms of exosomes [76–78]. Nanoparticle tracking analysis and dynamic light scattering provide size and distribution data via light scattering and Brownian motion analysis, with centrifuged exosomes generally falling between 30 and 120 nm in diameter.

Identifying specific EV markers is crucial for distinguishing subtypes and determining their origin. Techniques such as Western blot and flow cytometry, utilizing markers enriched in exosomes like CD63, Alix, and TSG101, facilitate this process. Detection of tissue-specific proteins, such as the glutamate transporter 1 (GLT-1) in astrocytes, helps trace EV sources. For instance, Zhang’s team used magnetic beads coated with antibodies against GLT-1 to isolate astrocyte-derived EVs for Parkinson’s disease diagnosis [79].

Biochemical analyses, including polymerase chain reaction and sequencing, are essential for assessing exosomal nucleic acids. Immunoassays and mass spectrometry-based proteomics provide insights into protein composition, further elucidating the functionality of EVs [68].

## Therapeutic potential of EVs in ischemic stroke

### Neuronal survival

Ischemic stroke initiates both programmed and unprogrammed neuronal death through multiple pathways, including apoptosis, pyroptosis, and ferroptosis, especially in the penumbral areas where neurons are potentially salvageable. The interplay between autophagy and cell death mechanisms complicates the landscape of neuronal survival [29, 80]. Inhibiting post-stroke apoptosis is crucial for functional recovery. For example, Xie et al. discovered that EV-miRNA-22-3p from adipose-derived stem cells (ADSCs) could suppress neuronal apoptosis in middle cerebral artery occlusion (MCAO) rat models and rat primary cortical neurons subjected to oxygen-glucose deprivation/reperfusion (OGD/R) by down-regulating histone demethylase KDM6B [81]. Inflammasome activation, particularly NLRP3, leads to caspase-1 activation and pyroptosis in AIS [82, 83]. Earlier research indicated that bone marrow stem cells (BMSCs)-derived exosomes shifted microglia toward an anti-inflammatory phenotype (M2), thereby mitigating neuronal pyroptosis [84]. The role of autophagy in stroke and how stem cell-derived exosomes influence this process remains debated, with evidence pointing to both neuroprotective and neurodestructive outcomes [85, 86]. Doeppner et al. argued that EVs from ADSCs offered neuroprotection and improved neurological function by inhibiting ischemia-induced autophagy [87]. Conversely, another study reported that BMSCs-derived exosomes activated AMPK-dependent autophagy and attenuated pyroptosis in PC12 cells. When this autophagy was inhibited by si-AMPK, the neuroprotective effect of exosomes against pyroptosis was diminished [88]. These discrepancies may be associated with differences in stem cell sources and experimental models. Ferroptosis, driven by intracellular iron accumulation and lipid peroxidation, is another target for intervention [29], with ADSCs-derived exosomes containing miRNA-760 showing promise in inhibiting ferroptosis and enhancing neurological function [78]. Addressing cellular death pathways and clarifying the role of exosomes are essential for developing novel therapeutic strategies (Table 2; Fig. 3D).

**Table 2:** Overview of specific molecules, pathways, and mechanisms involved in EV treatment for AIS

Clinical Scenario	Content	Model	Origination	Target or Pathway	Effect	Reference
AIS	miR-22-3p	MCAO-SD rats; OGD-primary cortical neurons	ADSCs	KDM6B/BMP2/BMF axis	Anti-apoptotic effects on neurons	[81]
AIS		MCAO-SD rats; OGD-BV2 and PC12 culture	BMSCs		M2-microglia polarization; Anti-pyroptosis of neurons	[84]
AIS	miR-25-3p	MCAO-C57BL/6 mice; OGD-primary neurons	ADSCs	p53/BNIP3	Anti-autophagy; Promotion of neuronal survival	[87]
AIS		OGD-PC12 cell	BMSCs	AMPK/mTOR	Activation of autophagy; Anti-pyroptosis	[88]
AIS	miR-760-3p	MCAO-C57/BL6 mice; OGD-N2a-neuroblastoma cell	ADSCs	miR-760-3p/CHAC1	Anti-ferroptosis	[78]
AIS		MCAO-aged C57BL6/j mice	BMSCs		Inhibition of leukocyte infiltration	[92]
AIS	LncRNA-KLF3-AS1	MCAO-C57BL/6J mice; OGD-BV2 microglia	BMSCs	LncRNA-KLF3-AS1/miR-206/USP22/Sirt1	Anti-inflammation	[93]
AIS	ZEB1	OGD-BV-2 microglia	NSCs	ZEB1/GRP30/TLR4/NF-κB	Anti-inflammation	[94]
AIS	miR-223-3p	MCAO-SD-rats; NMLTC_4_ stimulated BV-2 microglia	BMSCs	miR-223-3p/CysLT_2_R	Microglia phenotype conversion	[97]
AIS	miR-145	MCAO-SD-rats; OGD-BV-2 microglia	BMSCs	miR-145/FOXO1	Microglia phenotype conversion	[98]
AIS	miR-126	MCAO-SD rats; OGD-BV-2 microglia	ADSCs		Anti-inflammation; Pro-angiogenesis; Pro-neurogenesis;	[99]
AIS		MCAO-CB57/B6 mice; OGD-primary astrocytes	NSCs		Astrocyte protection	[101]
AIS	miR-138-5p	MCAO-C57BL/6 mice; OGD-treated primary astrocytes	BMSCs		Astrocyte protection	[103]
AIS	miR-125b-5p	MCAO-C57BL/6 N mice; OGD-treated primary astrocytes; Astrocyte-endothelia coculture model	hUC-MSCs	miR-125b-5p/TLR4/NF-*κ*B	Inhibition of astrocyte activation; BBB protection	[77]
AIS	Egr2	MCAO-C57BL/6 mice; OGD-bEnd.3 endothelial cells	BMSCs	Egr2/SIRT6-Notch1	Pro-angiogenesis	[106]
AIS	miR-21-5p	MCAO-SD rats; HUVECs	BMSCs		Pro-angiogenesis	[107]
AIS	miR-181b-5p	MCAO-Wistar rats; OGD-BMECs	ADSCs	miR-181b/TRPM7	Pro-angiogenesis	[108]
AIS	miR-26a	MCAO-SD rats; ODG-Primary NSCs from SD rats	USCs	miR-26a/HDAC-6	Pro-neurogenesis	[46]
AIS	miR-17-92 cluster	MCAO-Wistar rats	BMSCs	miR-47-92 cluster/PTEN/PI3K/Akt/mTOR/GSK-3β	Enhancement of neuroplasticity	[115, 116]
AIS	miR-124	MCAO-C56BL/6 mice	BMSCs		Pro-neurogenesis	[117]
AIS	miR-134	OGD-primary OLs	BMSCs		Anti-apoptotic effects on OLs	[120]
AIS	miR-128-3p	MCAO-C57BL/6 mice; Fibrinogen-treated-OPCs and OGD-OPCs	NSCs	miR-128-3p/BMP signaling pathway	Promotion of the maturation of OPCs into OLs	[122]

Abbreviations EV **=** extracellular vesicle; AIS = acute ischemic stroke; miR = microRNA; MCAO = middle cerebral artery occlusion; SD = Sprague-Dawley; OGD = oxygen glucose deprivation; ADSCs = adipose-derived stem cells; BMSCs = bone marrow stem cells; NSCs = neural stem cells; hUC-MSCs = human umbilical cord-derived mesenchymal stem cells; USCs = urine-derived stem cells; OLs = oligodendrocytes; OPCs = oligodendrocyte progenitor cells; BBB = blood-brain barrier

### Anti-neuroinflammation

Acute stroke rapidly activates and recruits peripheral immune cells and resident glial cells, which may exacerbate BBB leakage and neuronal death [23, 89]. Strategies to mitigate the influx of immune cells may reduce neuroinflammation in the acute stage of stroke [90, 91]. Hermann et al. demonstrated that intravenous administration of MSCs-derived EVs significantly diminished leukocyte infiltration into the ischemic region in MCAO mice, particularly affecting polymorphonuclear neutrophils, macrophages, and monocytes [92].

During brain injury, resident microglia not only remove cellular debris but also orchestrate the restorative process. Their polarization into distinct phenotypes (M1 and M2) at different stages may explain their dual roles. M1 microglia release proinflammatory cytokines, while M2 exert anti-inflammatory effects and secrete trophic factors. Thus, targeting microglial overactivation in the acute phase is a potential therapeutic strategy. Zhou’s team found that Exo-LncRNA-KLF3-AS1 from BMSCs inhibited ROS production, apoptosis, and the expression of inflammatory factors (MCP-1, TNF-α, IL-6) by binding miRNA-206 and stabilizing Sirt1 in OGD-treated BV-2 microglia [93]. Similarly, NSCs-released exosomes lowered the production of inflammatory agents (IL-1β, IL-6, TNF-α) in ODG-treated BV-2 microglia by delivering ZEB1, which upregulated GPR30 and inhibited the TLR4/NF-κB pathway [94]. The shift towards the M2 phenotype supports angiogenesis, axonal regeneration, synaptic plasticity, and white matter integrity after stroke [95]. The microenvironment dictates microglial phenotypes, with EVs from stem cells acting as potent signals for these shifts [96]. In MCAO rats and/or NMLTC_4_ stimulated BV-2 cells, exosomal miRNA-223-3p not only reduced pro-inflammatory cytokine expression (IL-1β, IL-6, TNF-α) but elevated IL-10 levels, facilitating the phenotypic transition of M1 microglia (CD16/32^+^) to M2 (CD206^+^) [97]. Additionally, exosomal miRNA-145 ameliorated OGD-induced injury in BV-2 microglia and promoted the shift from M1 (CD86^+^) towards M2 (CD206^+^) by down-regulating FOXO1 [98]. The immunomodulatory effects of EVs facilitate various repair processes, with miRNA-126-OE exosomes from ADSCs inhibiting microglial activity and inflammatory responses, and enhancing vWF and DCX expression in the peri-infarct zone of MCAO rats, highlighting the multitarget capabilities of EVs and their immunomodulatory impact [99] (Table 2; Fig. 3D).

### Astrocytes and BBB Protection

Astrocytes, the most abundant cell type within the central nervous system, are active participants in synaptic regulation, neuronal homeostasis maintenance, and BBB formation. Traditionally viewed as detrimental when activated post-OGD due to their release of inflammatory mediators, recent insights suggest that enhancing astrocyte survival and curbing reactive astrogliosis post-stroke may be pivotal for neuronal safeguarding and BBB preservation [100]. NSCs-derived exosomes markedly reduced lactate dehydrogenase release from OGD-treated astrocytes, correlating with reduced apoptosis as indicated by propidium iodide/Hoechst co-staining [101]. Lipocalin-2, secreted by active astrocytes, is known to exacerbate post-stroke inflammation and neurological deficits [102]. Fascinatingly, miRNA-138-5p, conveyed by BMSCs-derived exosomes, substantially suppressed lipocalin-2 secretion, attenuating astrocyte apoptosis and thereby enhancing neuronal protection [103]. Moreover, the challenge of hemorrhagic transformation during IVT with tPA, especially outside the optimal therapeutic window, underscores BBB disruption as a principal pathological factor. Research by Wang et al. revealed that EVs from human umbilical cord MSCs could mitigate tPA-induced BBB disruption both in vivo and in astrocyte-endothelial co-culture models. These EVs facilitated the delivery of miRNA-125b-5p to astrocytes, targeting the TLR4/NF-*κ*B signaling pathway and thus maintaining BBB integrity [77]. Therefore, MSCs-EVs represent a potential adjunct therapy for BBB repair and prevention of IVT-associated complications (Table 2; Fig. 3D).

### Angiogenesis and vascular remodeling

The link between enhanced microvasculature in peri-infarct regions and improved neurological function highlights the significance of angiogenesis in stroke recovery [104]. Thus, promoting angiogenesis through pharmacological or molecular interventions is a compelling avenue for research. Emerging evidence indicates that stem cell-derived EVs can modulate AIS progression through angiogenic mechanisms. Particularly, the synergistic action of the Notch and VEGF-VEGFR pathways in brain collateral vessel angiogenesis has been well-documented [105]. Xia’s team reported that exosomes from BMSCs encouraged endothelial tube formation in OGD-exposed bEnd.3 cells by delivering Egr2, which enhanced SIRT6 expression and subsequently suppressed Notch1 [106]. Moreover, these exosomes significantly increased the number of BrdU+/vWF + cells and the expression of angiogenic markers (VEGF, VEGFR2, Ang-1, Tie-2), thereby alleviating brain injury in MCAO models via miRNA-21-5p upregulation [107]. Similarly, ADSCs-derived exosomes boosted endothelial cell migration and tube formation, with the overexpression of transient receptor potential melastatin 7 mitigating the effects of miRNA-181b [108]. These findings illuminate the potential of exosomes to drive angiogenesis, pivotal not just for re-establishing blood flow but also for laying the foundation for neurogenesis and axonal outgrowth [109] (Table 2; Fig. 3D).

### Neurogenesis

The discovery of adult neurogenesis, particularly in the SVZ of the lateral ventricle and the SGZ of the dentate gyrus [110–113], marks a significant breakthrough in understanding post-stroke brain recovery mechanisms. Activated NSCs in these areas proliferate, migrate to the peri-infarct zone, and differentiate into mature neurons to repair the damaged neural network [114].

EVs from MSCs and NSCs have shown therapeutic potential in AIS by enhancing neurogenesis and neuroplasticity. Deng et al. demonstrated that exosomes from human urine-derived stem cells (USCs) reduced infarct volume and improved neurological function in MCAO rats. Treatment with USC-Exos significantly increased NSC proliferation in the SVZ, as evidenced by enhanced EdU+/Nestin + or EdU+/Sox2 + staining, and a rise in Tuj-1 + cells [46]. Beyond neurogenesis, regulating neuroplasticity through exosome mediation is crucial for neuronal recovery [91], with miRNA-17-92 cluster-enriched exosomes boosting axonal-myelin bundle density, dendritic plasticity, and synaptic plasticity by down-regulating PTEN and deactivating GSK-3β [115]. These tailored exosomes also enhanced transcallosal axonal plasticity and promoted contralesional axonal sprouting into the denervated spinal cord, thereby aiding in the rewiring of spinal motor neurons after stroke [116]. Additionally, Yang’s team pioneered the use of modified exosomes, incorporating rabies virus glycoprotein (RVG) and a plasmid overexpressing miRNA-124, to boost neurogenesis and reduce brain injury [117]. Remyelination, essential for neurological recovery [118, 119], focuses on protecting oligodendrocytes, which are susceptible to hypoxia, neuroinflammation, and oxidative stress following stroke. Enhancing oligodendrocyte survival and promoting oligodendrocyte regeneration from oligodendrocyte progenitor cells (OPCs) are key strategies for augmenting remyelination. Zhu’s team highlighted the use of BMSCs-derived miRNA-134 to prevent oligodendrocyte apoptosis by targeting caspase-8 [120]. Fibrinogen leakage from the compromised BBB can activate the BMP signaling pathway in OPCs, impeding remyelination [121]. Remarkably, Exosomes from NSCs were found to elevate myelin basic protein levels and inhibit the BMP pathway by delivering miRNA-128-3p to fibrinogen-exposed OPCs [122]. Based on the evidence presented, stem cell-derived exosomes may serve as effective candidates for promoting neurogenesis and neuroplasticity after cerebral ischemia (Table 2; Fig. 3D).

## Approaches to enhance the efficacy of EVs

Although stem cell-derived exosomes offer considerable promise in ameliorating stroke pathophysiology, their therapeutic efficacy in vivo is limited by challenges such as suboptimal targeting, diminished therapeutic potency, and rapid elimination from the bloodstream. Enhancing these attributes with the assistance of biomedical engineering is therefore indispensable. A key goal in EV engineering is to produce vesicles enriched with therapeutic agents and capable of precise targeting. This section delves into various engineering approaches designed to augment the functionality of EVs for stroke therapy, with a focus on targeting modifications and cargo enrichment.

### Targeting modification

**Fig. 4:**
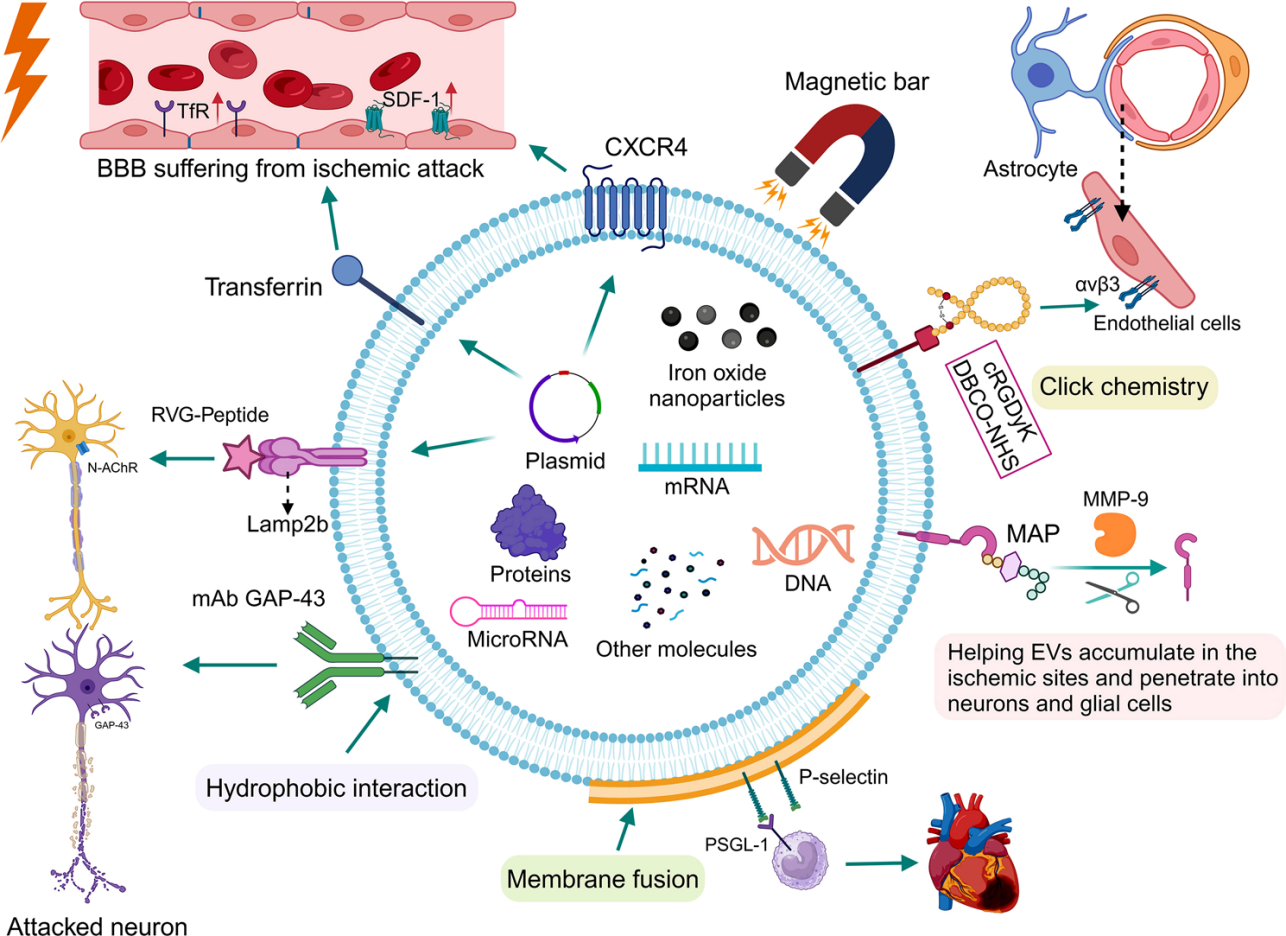
Targeting modification for EVs. Leveraging the increased expression of specific biomolecules within NVUs after a stroke, techniques such as hydrophobic insertion, gene editing, chemical conjugation, and membrane fusion are used to express functional ligands on the surface of EVs, enhancing their homing to NVUs and ischemic brain tissue. Additionally, physical targeting methods, facilitated by the interaction between IONP inside EVs and external magnetic fields, also demonstrate efficacy. Abbreviations: EVs = extracellular vesicles; NVUs = neurovascular units; IONP = iron oxide nanoparticles; MAP = matrix metalloproteinases-activatable peptides. Figure created with BioRender.com

Given the low targeting property of unmodified EVs, most vesicle-based bioactive drugs fail to reach ischemic sites and are rapidly cleared, resulting in suboptimal efficacy and significant EV wastage. To overcome this, engineering EVs with improved targeting capabilities have involved the addition of various molecules, such as fusion proteins, cyclopeptides, monoclonal antibodies, and nanoparticles, into the surfaces and interiors of EVs (Fig. 4). A notable innovation was the conjugation of the RVG peptide and the Lamp2b protein, which created a molecule with enhanced central nervous system targeting potential. Experimental data showed that RVG-Lamp2b-modified exosomes preferentially accumulated in ischemic brain regions in mice, showcasing specific localization to lesion sites [117]. Additionally, ischemia and hypoxia prompt differential expression of certain membrane proteins in the brain, offering opportunities to utilize these proteins and their ligands (both natural and synthetic) to improve EV targeting. Inspired by the upregulated transferrin receptor expression on endothelial cells post-ischemia, Li’s team developed a novel approach by increasing the density of transferrin on EV surfaces for targeted accumulation [123, 124]. Likewise, under hypoxic conditions, the expression and release of SDF-1 increase with the induction of integrin kinases and HIF, which play significant roles in later neurogenesis and angiogenesis through the SDF-1/CXCR signaling pathway [125, 126]. Compared to undecorated EVs, treatment with EVs from CXCR4-overexpressing MSCs resulted in improved neurological outcomes and reduced infarct volumes [127]. However, the variability in targeting peptide stability and potential degradation during EV synthesis necessitates further empirical validation.

Beyond natural membrane proteins, synthetic polypeptides, antibodies, and pro-peptides present promising alternatives for enhancing EV targeting capabilities. Following ischemic events, the expression of integrin αvβ3 on cerebral vascular endothelia increases, making it an ideal target for engineered EVs [128]. Recent advancements in bioconjugation techniques, such as copper-free click chemistry, have enabled the efficient attachment of longer peptides and proteins to EVs, with RGD-exo showing improved affinity towards ischemic regions [129]. Furthermore, monoclonal antibodies targeting specific neuronal proteins (e.g., GAP-43) and pro-peptides (e.g., MMP-activatable peptides) activated by stroke-induced enzymes have demonstrated enhanced targeting and therapeutic outcomes [59, 130]. The integration of membrane components from other sources, such as platelets or liposomes, through membrane-fusion techniques, endows EVs with characteristics inherent to their donor cells. This is exemplified by engineered platelet-mimetic EVs, which were rapidly engulfed by macrophages in the infarcted area due to P-selectin and PSGL-1 interaction, thereby exerting immunomodulatory effects to extinguish excessive inflammatory responses [131]. The advent of magnetic nanovesicles, bioengineered with iron oxide nanoparticles, heralds a novel direction for targeted EV delivery, using external magnetic fields to guide EVs to ischemic sites [132]. Nonetheless, the safety of such modifications, especially concerning stroke-induced ferroptosis from iron overload, warrants thorough evaluation. In essence, preserving the structural integrity of EVs across these strategies is paramount, ensuring effective BBB crossing and cell internalization. Ultimately, the pursuit of safe, efficient, and practical targeting modalities will significantly bolster the therapeutic utility of EVs in stroke management.

### Cargo Modification

EVs, serving either as therapeutic agents or drug delivery vehicles, require precise modification and cargo enrichment to achieve optimal efficacy. Modification techniques are broadly classified into pre-isolation and post-isolation strategies, each chosen based on specific goals and application contexts, accompanied by a careful analysis of their respective benefits and limitations.

#### Pre-isolation modification

**Fig. 5:**
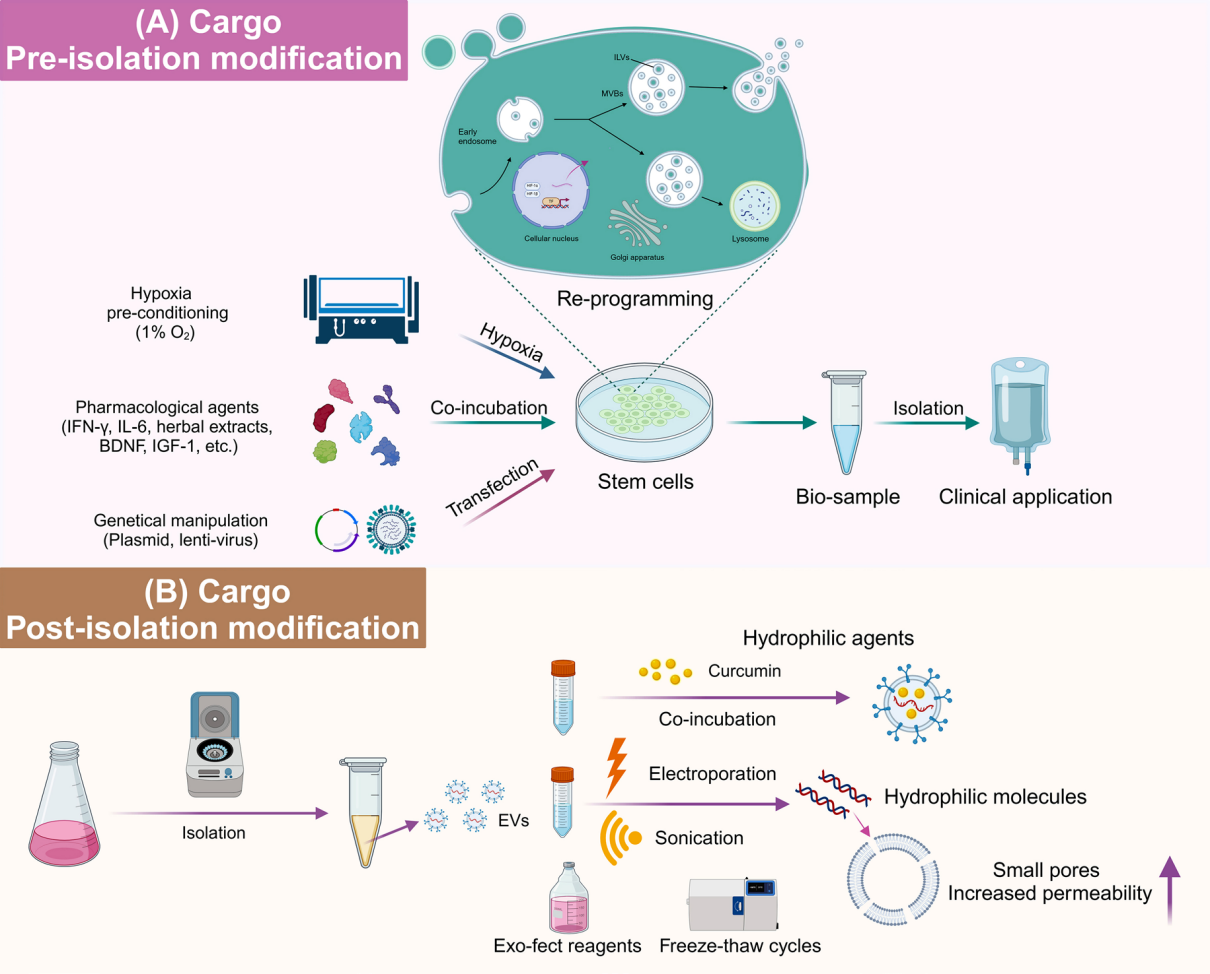
Approaches of cargo modification for EVs. **(A)** Pre-isolation cargo modification: This involves preconditioning parental stem cells using specific interventions to induce metabolic changes that influence the molecular cargo of the resultant EVs. Strategies like hypoxic preconditioning, treatment with pharmacological agents or cytokines, and genetic modification are among the commonly employed precursory modifications. **(B)** Post-isolation cargo modification: This technique entails the direct modification of EVs after their isolation from stem cell cultures or biological fluids. Hydrophobic drugs can integrate into EVs through simple diffusion across the lipid bilayer. Conversely, introducing hydrophilic compounds into EVs may require techniques to enhance EV membrane permeability, such as electroporation, sonication, the use of transfection agents, or repeated freeze-thaw cycles to facilitate entry. Abbreviations: EVs = extracellular vesicles; IFN-γ = interferon-γ; IL-6 = interleukin-6; BDNF = brain-derived neurotropic factor; IGF-1 = insulin-like growth factor

Pre-isolation modification involves strategic alterations of parent stem cells to modify their exosomal secretion profiles or enrich specific therapeutic molecules. This approach, while relatively simple and conducive to preserving EV membrane integrity, may introduce variability due to the complex nature of EV biogenesis. Preconditioning, or metabolic reprogramming of donor cells, is achieved through modifications to the culture environment, employing strategies such as hypoxic preconditioning, cytokine stimulation, pharmacological pre-treatment, genetic manipulation, and co-incubation (Fig. 5A).

##### Hypoxia Preconditioning

Hypoxia preconditioning is a widely adopted technique for modifying stem cells and their EVs [133]. Under low-oxygen conditions, HIF-α stabilizes and forms a heterodimer with HIF-β to activate hypoxia-responsive genes. Critical molecules such as VEGF/VEGFR, Rabep2, Notch ligand (DLL-4 and Jagged), IGF-2, TGF-β, and miRNA-210 are pivotal in this process [134, 135]. Liu et al. illustrated that hypoxia-preconditioned MSCs significantly surpass their naive counterparts in enhancing pial collateral remodeling, improving cerebral blood flow, and increasing vascular density within the peri-infarct zone, with Rabep2 upregulation playing a key role [136]. EVs from cells conditioned under hypoxia have shown enhanced therapeutic potential, reflecting the hypoxic adaptations of their source cells. EVs from BMSCs cultured under 1% O_2_ conditions showed dose-dependent improvements in the proliferation, migration, and tube formation of hCMEC/D3 cells, as well as promoting cell survival under OGD conditions. Moreover, mice treated with hypoxia-conditioned EVs exhibited better neurological outcomes, smaller infarct volume, and greater vascular and neuronal presence in affected striatal regions 56 days post-stroke [137]. Li et al. observed similar benefits with EVs from umbilical cord-derived MSCs pretreated with extracts from infarcted brain tissue, leading to pronounced vascular remodeling and neurological improvements in MCAO rats [138]. Additionally, EVs from ADSCs cultured in hypoxia substantially reduced pro-inflammatory cytokine levels and prompted a shift in microglia from the M1 to M2 phenotype in the ischemic hippocampus [139].

##### Cytokine and pharmacological agents pre-treatment

Despite the recognized safety and feasibility of stem cell transplantation for ameliorating neurological deficits, the survival rate of transplanted stem cells in the harsh post-stroke microenvironment is low [140]. Researchers have conceived various engineering methods to enhance the resilience and environmental modulation capability of stem cells, including cytokine pretreatment. For instance, co-injecting NSCs with IFN-γ at the ischemic site significantly boosted neurogenesis and angiogenesis in the peri-infarct area of MCAO rats [141]. IL-6 pre-exposure reprogrammed NSCs to resist oxidative damage by activating STAT3 [142]. These findings underscore the importance of cytokine-preconditioned stem cells in augmenting the therapeutic effects of their secreted EVs. In a comparative study, EVs from IFN-γ-preconditioned NSCs were notably more effective in supporting neuronal survival, stimulating neurogenesis and angiogenesis, and inhibiting M1 microglia activation, leading to improved neurological function [143].

Buyang Huanwu Decoction (BYHWD), composed of seven traditional Chinese herbs, is renowned for its neuroprotective properties [144], particularly in reducing neuroinflammation and promoting vascular regeneration in AIS [145–147]. Che’s research on combining BYHWD with MSC transplantation revealed that EVs from MSCs and NSCs preconditioned with BYHWD increased vascular density and upregulated VEGF and Ki-67b expression in brain tissues, a process partly mediated by miRNA-126 [145, 148]. Further investigation into the impact of BYHWD on NSC-EVs showed a significant increase in EV secretion, boosting differentiation into MAP-2 + neurons and GFAP + astrocytes both in vitro and in vivo [149]. This underscores the potential of integrating traditional Chinese medicine extracts and cytokine preconditioning to customize EV cargo, maximizing therapeutic efficacy for post-stroke neurological recovery and paving the way for developing potent EV-based therapeutics.

##### Genetic manipulation

Gene editing techniques are increasingly employed to enhance the therapeutic efficacy of EVs. Utilizing lentiviral vectors or plasmids to precisely modulate the expression of specific RNAs provides substantial benefits. Prime targets for genetic engineering include transcription factors, neurotrophic factors, lncRNAs, and miRNAs integral to stroke pathophysiology. Such genetic manipulation initiates a cascade of biochemical reactions, altering the transcriptional and translational profiles of cells and their EVs. For example, transfecting MSCs with a recombinant lentivirus containing the HIF-ɑ gene markedly upregulated Jagged-1 expression on EV surfaces, subsequently potentiating their angiogenic effects [150]. Brain-derived neurotrophic factor (BDNF), known for its neuroprotective efficacy across a spectrum of neurological conditions [151], when loaded into MSC-derived EVs, yielded improvements in functional behavior and neurovascular unit reconstruction through the BDNF/TrkB signaling pathway [152]. Similarly, MSCs overexpressing the lncRNA KLF3-AS1 released EVs that exhibited enhanced neuroprotective effects by stabilizing Sirt1 [93]. These examples illustrate the versatility and potential of gene editing in refining the therapeutic capabilities of EVs, with future research poised to uncover additional targets for genetic manipulation.

#### Post-isolation modification

Post-isolation modification directly alters isolated and purified EVs, enabling the incorporation or expression of targeted molecules on the EV surface. This approach offers two main advantages: it operates independently of the biogenesis mechanism of EVs, circumventing the “black-box effect” and producing more uniform EVs; and it serves as an alternative for EVs derived from complex sources like serum, where parent cells are varied and arduous to obtain. However, challenges include complex processing, separation difficulties, and potential EV membrane damage **(**Fig. 5B**)**.

Common methods for post-isolation modification or exogenous drug loading encompass drug incubation, electroporation, low-frequency ultrasound, and repeated freeze-thaw cycles. For hydrophobic drugs, co-incubation with EVs under hydrophobic conditions allows passive diffusion into the vesicles. Gao et al. demonstrated this by successfully encapsulating curcumin in exosomes using PBS [153]. However, simple co-incubation often results in suboptimal loading efficiency, spurring the development of active loading methods utilizing auxiliary approaches, such as Exo-fect reagents, low-frequency ultrasound, electroporation, and repeated freeze-thaw cycles [154]. Chen et al. reported improved anti-inflammatory and neuro-regenerative effects in NSC-EVs by using Exo-fect reagents to incorporate BDNF, highlighting the effectiveness of these strategies [155]. Ou et al. employed sonication to encapsulate Tβ4 into EVs, showcasing the delicate balance between increasing loading efficacy and maintaining EV integrity [156]. Although active loading techniques can enhance efficiency, they may compromise EV integrity, risking vesicle rupture or fusion, thus impacting their biocompatibility and biological functionality. Therefore, selecting an appropriate loading strategy that considers the drug’s physicochemical properties is crucial for optimizing both efficiency and safety. More details about the advantages and disadvantages of each modification technique are listed in Table 3.

**Table 3:** Summary of pre- and post-isolation techniques for EV modification

Techniques	Detailed conditions	Effects	Pre- or post-isolation	Advantages	Disadvantages
Hypoxia pre-conditioning	Stem cells cultured in hypoxic conditions for 24–48 h	Higher concentration of therapeutic miRNAs and proteins in EVs, especially those related to angiogenesis	Pre-isolation manipulation	High safety (low immunogenicity and tumorigenicity); Minimal impact on EV structure and viability; Flexibility and plasticity; Diversity and multifunctionality	Difficult to achieve large-scale production; Poor stability; Prone to degradation during storage and transportation; High heterogeneity (black-box effect)
Cytokine or pharmacological agent treatment	Stem cells pre-treated with cytokines (IFN-γ, IL-6) or specific drugs (BYHWD)	Occurrence of re-programming in stimulated stem cells, leading to the secretion of EVs with enhanced therapeutic potential (e.g., miRNA-126)			
Genetic editing	Transfection of recombinant lentivirus or plasmid into stem cells	Elevating the quantity of certain molecules in EVs through increasing their expression in stem cells			
Co-incubation	Mixing the purified EVs with certain drugs and incubating in a thermostatic shaker for a period of time at room temperature	Pharmacological agents diffusing into the EVs following a concentration gradient	Post-isolation manipulation	High drug-loading capacity; Low toxicity; Strong controllability; Low cost	Poor storage stability; More suitable for hydrophobic drugs (e.g., curcumin)
Electroporation	Under the help of transient electric current pulse to temporarily enlarge the pores on EV membranes	Pharmacological agents diffusing into the EVs through the enlarged pores on EVs		Strong controllability; High efficiency; Wide applicability	Requirement for specialized equipment; Potential safety concerns
Sonication	Under the help of pulsed and focused ultrasound technology to temporarily enlarge the pores on EV membranes			Strong controllability; Wide applicability	Damage to the structure or activity of EVs
Transfection agents	With the assistance of chemical agents to allow specific DNA or RNA fragments to enter EVs			High purity; High stability	Complex process; Difficult to control; Time-consuming and labor-intensive
Freeze-thaw cycles	Mixture of EVs and drugs, rapidly freezing at -80℃ and thawing at RT, repeating 3–5 times			Simple; Environmentally friendly	Low production efficiency; Limited applicability; Membrane damage

Abbreviations EVs = extracellular vesicles; IFN-γ = interferon-γ; IL-6 = interleukin-6; BYHWD = Buyang Huanwu Decoction; miRNA = microRNA; RT = room temperature

## Scalable and effective manufacture of EVs

The promise of stem cell-derived EVs in treating neurological disorders is unmistakable, yet their clinical application is constrained by the lack of technologies for large-scale, uniform production [56]. To surmount this obstacle, innovations such as exogenous drug stimulation [157], transient electrical stimulus [158], gene editing [150, 159], hypoxia preconditioning [160], and three-dimensional (3D) cell culture have been explored to augment EV yield. Among these, 3D cell culture has gained considerable attention due to its ability to ensure batch-to-batch consistency, adherence to Good Manufacturing Practice guidelines, and stringent quality control [161].

In contrast to traditional two-dimensional flasks, which confine stem cells to a planar surface and may impede intercellular communication, 3D culture platforms such as micropatterned wells, hydrogel scaffolds from 3D bioprinting (3DBP), bioreactors, and organoids offer enhanced environments. Oh Young Bang’s team employed a micropatterned well system for MSC spheroid formation, achieving not only high yields but also consistent EV properties across batches. These EVs notably enhanced neurological recovery and reduced infarct volumes in animal models, validating the effectiveness of this method [162]. Furthermore, EVs from this platform have proven beneficial in other disease models, such as wound healing, where they facilitate critical healing stages, such as re-epithelization, granulation tissue maturation, and new blood vessel growth [163].

Recently, 3DBP has emerged as a method for creating complex scaffolds that mimic biological niches, facilitating stem cell interactions and signal transmission [164]. Xin et al. used gelatin methacryloyl hydrogel scaffolds for MSC culture to explore the therapeutic effects of the resulting EVs in treating AIS [165]. The EVs from 3D cultures showed superior ischemic targeting and microglial endocytosis, alongside enhanced neovascularization and anti-inflammatory effects. Additionally, the use of Vertical-Wheel bioreactors demonstrated a 2.5-fold increase in human-MSC EV secretion, resulting in alterations in their miRNA and protein content which may underpin improved therapeutic outcomes [166]. The advancement of 3D culture technologies marks a critical step towards the clinical translation of EVs, offering a scalable, efficient, and quality-controlled production method.

## Innovative combination with other treatments

**Fig. 6:**
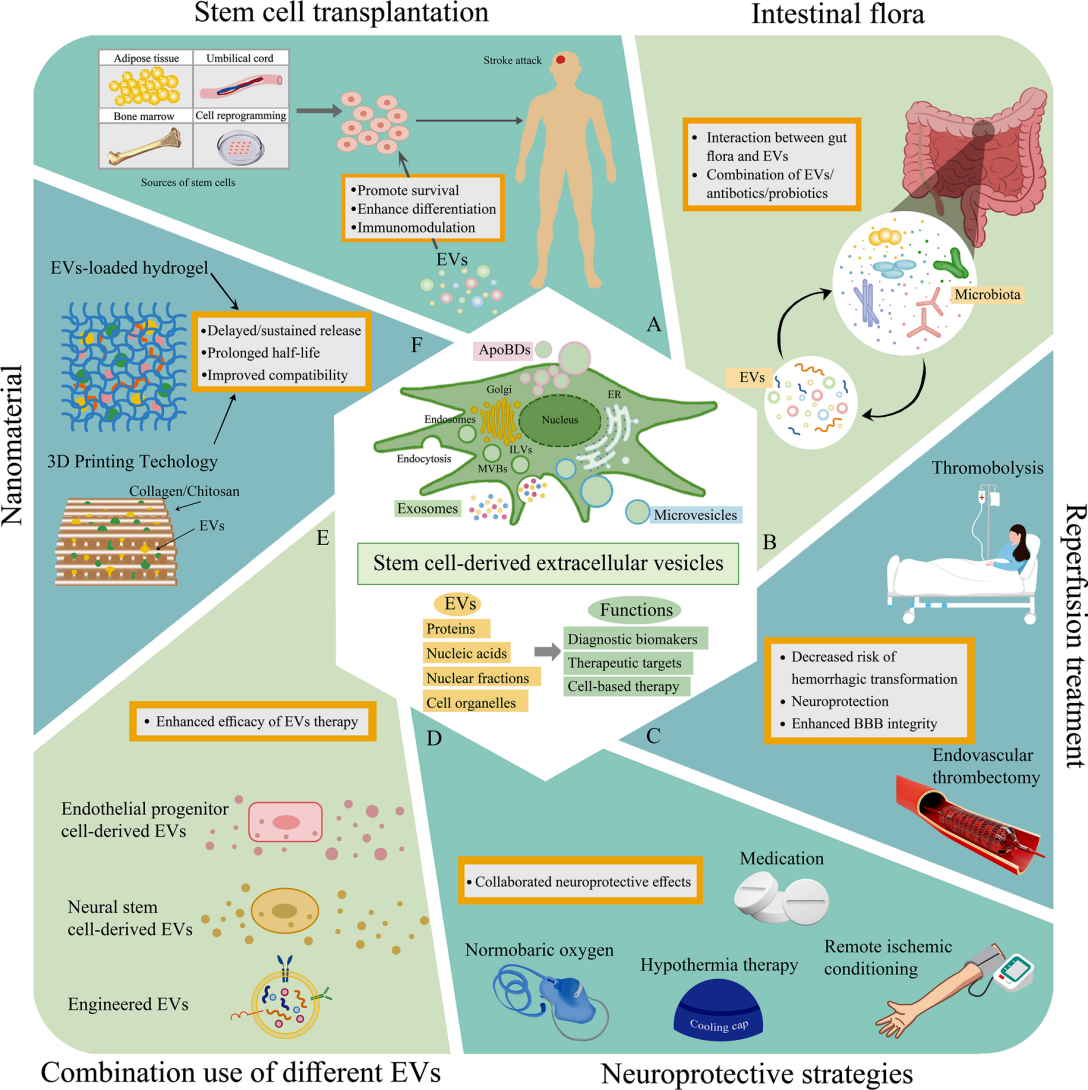
EVs in conjunction with various other therapeutic strategies. **(A)** With stem cell transplantation: Applying EVs to the infarcted lesion before introducing stem cells can improve the lesion environment, thus enhancing subsequent stem cell survival and differentiation. **(B)** With intestinal flora: Using antibiotics or probiotics to regulate the intestinal microbiota may improve the therapeutic effects of EVs in treating brain disorders. **(C)** With reperfusion therapy: EVs can serve as potential neuroprotective agents, extending the time window for reperfusion therapy and reducing the risk of hemorrhagic transformation. **(D)** With other neuroprotective interventions: Combining EVs with other neuroprotective methods could enhance overall effects, protecting the brain from damage in multiple ways. **(E)** With another type of EVs: Using one type of EV along with another from a different source could target a broader array of biological pathways and mechanisms. **(F)** With nanomaterials: Hydrogel or 3D-printed scaffolds can be used to encapsulate EVs, which allows for controlled and sustained release, enhances the stability and half-life of EVs, and improves tissue compatibility. Abbreviations: EVs = extracellular vesicles; 3D = three-dimensional; BBB = blood-brain barrier

Stem cell-derived EVs hold exceptional promise for cytoprotection in the acute phase of stroke and for neurovascular unit reconstruction during the subacute or chronic phases. However, as a standalone therapy, their efficacy is limited, necessitating the exploration of novel combination therapies, especially in the “reperfusion era” [167]. Following AIS, the adverse microenvironment and inflammatory reactions within the infarcted lesion have persistently impeded the efficacy and clinical translation of stem cell therapy. Pretreating MCAO/R mice with NSCs-EVs before transplantation can significantly reduce oxidative stress and inflammation in the infarct area, thereby regulating the local microenvironment and promoting the survival and differentiation of subsequently transplanted NSCs [168] (Fig. 6A).

Emerging evidence on the role of gut microenvironment modifications and gut-brain axis interactions in stroke pathophysiology opens new avenues for therapeutic intervention [169, 170]. Studies have indicated that EV administration can modulate the gut microbiota and metabolic profile. Intriguingly, Jiang et al. uncovered that in Alzheimer’s disease (AD) transgenic models, established gut microbiota and metabolite profiles could diminish the therapeutic impact of EVs on AD pathology [171] (Fig. 6B). This revelation prompts further investigation into whether modifying the gut microenvironment via antibiotics or probiotics could amplify the therapeutic benefits of EVs in treating stroke. Recent advancements in IVT [172–175] and EVT [176–178] have established them as cornerstone treatments for AIS. Integrating cytoprotective agents like EVs with these reperfusion strategies offers a synergistic opportunity to enhance therapeutic outcomes [179]. Wu et al. demonstrated that MSC-EVs could preserve BBB integrity after tPA administration, thus reducing the risk of hemorrhagic transformation associated with IVT [77] (Fig. 6C). Nonetheless, most preclinical studies to date have focused on MCAO/R animal models without combining EVs with IVT or EVT treatments.

Additionally, non-pharmacological neuroprotective strategies, such as remote ischemic conditioning (RIC) [180, 181], hypothermia [182], electroacupuncture (EA) stimulation [183], and physical exercise [184, 185], have also shown effectiveness in neuroprotection and neural function restoration after stroke. RIC, a safe and effective treatment for AIS [186, 187], may alter plasma EV profiles [188, 189], suggesting its potential as a complementary therapy with EVs. Similarly, EA in combination with iPSC-derived EVs displayed synergistic effects in reducing neuronal apoptosis and modulating microglial activation via the IL-33/ST2 axis [190]. Exercise, when coupled with NSC-derived EVs in MCAO models, significantly suppressed neuronal apoptosis and promoted axonal regeneration and synaptic remodeling [191] (Fig. 6D).

Given the complex pathophysiology of stroke, therapies targeting a single pathway often fall short in providing adequate cytoprotection. Hence, combination therapy involving different types of EVs presents a rational strategy. Chen et al. discovered that using EVs from endothelial progenitor cells and EVs from neural progenitor cells together markedly reduced apoptosis and ROS production, surpassing the efficacy of either EV type alone [192] (Fig. 6E). Despite EVs’ low immunogenicity, their rapid clearance from the bloodstream poses a challenge [193]. Nanomedicine-based strategies aiming to stabilize EVs and ensure their controlled release are under exploration. Co-delivery platform systems utilizing biocompatible materials, such as hydrogels, have shown promise in extending EV half-life and improving delivery efficiency at injury sites [194] (Fig. 6F). This comprehensive approach emphasizes the multifaceted potential of EVs in improving stroke outcomes and underscores the need for further research and development in this promising field.

## Challenges and prospects

### Clinical Translation challenges

The therapeutic potential of stem cell-derived EVs for stroke treatment is promising, yet several obstacles must be addressed for successful clinical translation. A primary challenge is the scalability of EV production while preserving their intrinsic therapeutic properties. Current methodologies for the isolation and purification of EVs, such as ultracentrifugation and size-exclusion chromatography, are labor-intensive and inefficient at a clinical scale, highlighting the need for novel approaches that can cater to the high throughput demands inherent in stroke treatment. Additionally, targeting efficiency remains a critical hurdle, as the heterogeneity of ischemic brain environments necessitates precision in EV delivery to affected neuronal and vascular tissues. This precision targeting is paramount in stroke therapy, where the therapeutic window and localized delivery can significantly influence outcomes. Systemic or intravascular EV administration holds great promise for clinical translation. While targeting modification can increase the concentration of EVs in the ischemic lesion, the systemic effects of intravenous EV delivery—particularly the alteration of AIS pathophysiology through peripheral immunomodulation—cannot be overlooked. It is crucial to explore whether these remote systemic effects can improve the prognosis of AIS. In stroke patients, safety concerns, particularly regarding immunogenicity and oncogenicity, are of great importance due to the presence of comorbidities that may exacerbate potential adverse effects. A thorough understanding of the complex interplay between stem cell-derived EVs and the patient’s immune system, along with strategies to mitigate this risk, is essential to ensure safety in this vulnerable population. Additionally, the dosage of EVs warrants more attention. Determining the optimal dosage is of considerable clinical value, as it will not only maximize the therapeutic benefits of EVs but avoid potential side effects. The regulatory landscape for EV-based therapies is still in its infancy. Despite the establishment of regulatory frameworks for cell-based therapies by agencies such as the U.S. Food and Drug Administration and the European Medicines Agency, specific guidance tailored to the unique attributes of EVs is still evolving. The heterogeneity of EVs and the incomplete recognition of their therapeutic mechanisms in stroke further complicate their clinical application. Elucidating these mechanisms will provide valuable insights into how EVs can be manipulated for enhanced therapeutic outcomes.

### Strategies to overcome translational hurdles

To surmount these translational barriers, a multipronged strategy is imperative. First, the development of scalable, high-yield production methods for EVs is crucial, with bioreactor technologies and microfluidic systems representing promising avenues for the industrial-scale production of clinically applicable EVs. These technologies facilitate the controlled, reproducible generation of EVs to ensure consistency in quality and therapeutic efficacy. Second, advancements in nanotechnology and molecular engineering offer viable solutions to enhance the targeting specificity and delivery efficiency of EVs. Conjugation of EVs with peptides or antibodies that recognize ischemic brain tissue markers can significantly improve their homing capabilities. Innovative delivery methods, including intranasal routes, present non-invasive, efficient alternatives for crossing the BBB, potentially revolutionizing EV administration protocols. Third, addressing safety and regulatory concerns requires a robust framework for preclinical and clinical evaluation. Long-term studies to assess immunogenicity, tumorigenicity, and other potential adverse effects of stem cell-derived EVs are paramount. Developing non-invasive monitoring techniques to track EV distribution and persistence in vivo can aid in safety assessments. In parallel, regulatory bodies must establish clear, stroke-specific guidelines that facilitate the rapid yet safe development of EV-based treatments. Fourth, the identification of biomarkers reflective of stroke severity and responsiveness to EV therapy is another critical strategy. These biomarkers will enable patient stratification and the customization of treatment protocols, enhancing therapeutic outcomes. Lastly, fostering collaboration among researchers, clinicians, industry partners, and regulatory agencies is essential for advancing the translation of EV-based therapies. Sharing data and best practices can accelerate the development of standardized protocols and regulatory guidelines.

### Ongoing clinical trials

The clinical landscape is already witnessing the initiation of trials exploring the utility of stem cell-derived EVs in stroke management. Two phase I/II trials (NCT03384433 and NCT06138210) represent pivotal steps toward integrating EV-based therapies into stroke treatment paradigms. The results from these trials will not only offer insights into the safety and efficacy of EV therapies in stroke patients but also pave the way for further clinical investigations tailored to specific stroke subtypes and stages.

## Conclusion

This review underscores the potential of stem cell-derived EVs in revolutionizing AIS therapy, emphasizing their role in modulating the ischemic environment and enhancing recovery. Despite existing translational challenges, the integration of EVs into stroke treatment signifies a paradigm shift towards personalized regenerative medicine. As we navigate the complexities of bringing EV-based therapies from bench to bedside, their promise in improving stroke outcomes remains clear, marking a significant step forward in combatting this global debilitating condition.

## Data Availability

No datasets were generated or analysed during the current study.
